# Main Causes of Death in Piglets from Different Brazilian Nursery Farms Based on Clinical, Microbiological, and Pathological Aspects

**DOI:** 10.3390/ani13243819

**Published:** 2023-12-11

**Authors:** Jean Carlo Olivo Menegatt, Fernanda Felicetti Perosa, Anderson Hentz Gris, Manoela Marchezan Piva, Guilherme Carvalho Serena, Diego Luiz Bordignon, Carolina Reck, Álvaro Menin, Tatiane Terumi Negrão Watanabe, David Driemeier

**Affiliations:** 1 Setor de Patologia Veterinária, Faculdade de Medicina Veterinária, Universidade Federal do Rio Grande do Sul, Av. Bento Gonçalves, 9090, Porto Alegre 91540-000, RS, Brazil; fernandaperosa7@gmail.com (F.F.P.); anderson_gris@hotmail.com.br (A.H.G.); manoela.marchezan@gmail.com (M.M.P.); guilherme.serena@gmail.com (G.C.S.); ddriemeier@gmail.com (D.D.); 2 Cargill Alimentos Ltd.a., Av. José Bonifácio Coutinho Nogueira, 150, Campinas 13091-611, SP, Brazil; diego_bordignon@cargill.com; 3 VERTÀ Laboratórios, Instituto de Pesquisa e Diagnóstico Veterinário, Av. Lions, 1380—Nossa Senhora Aparecida, Curitibanos 89520-000, SC, Brazil; carolina@verta.vet.br (C.R.); alvaromenin@gmail.com (Á.M.); 4 Departamento de Biociências e Saúde Única, Universidade Federal de Santa Catarina, R. Germano A. Souza, Curitibanos 89520-000, SC, Brazil; 5 Department of Population Health and Pathobiology, College of Veterinary Medicine, North Carolina State University, Raleigh, NC 27606, USA; tati.terumi@yahoo.com.br; 6 Antech Diagnostics, West Olympic Blvd, Los Angeles, CA 90064, USA

**Keywords:** nursery pigs, mortality, food animals, infectious diseases, swine pathology, streptococcosis, bacterial polyserositis

## Abstract

**Simple Summary:**

Mortality within the nursery pig herd constitutes a pivotal factor contributing to economic losses and concurrently serves as a valuable indicator of production efficiency. The correct diagnosis is an important tool for guiding management corrections, improvements in animal performance, and decision making in herds. Thus, this study aimed to establish the main causes of death in nursery pigs from different nurseries through necropsies. Eighteen nurseries were analyzed (a total of 120,243 housed piglets), and 557 necropsies were performed. Conclusive diagnosis totaled 93.2% (519/557). Bacterial and viral diseases constituted the majority of conclusive diagnoses (72.9%) (378/519), followed by undefined causes at 14.4% (75/519) and non-infectious diseases at 12.7% (66/519). There was an increase in mortality rate in individual nurseries associated mainly with bacterial disease outbreaks. Therefore, infectious diseases constituted the largest portion of the diagnoses, offering a great opportunity for improving production rates in herds. In this sense, the aspects highlighted of the main causes of death can guide accurate diagnosis and specific decision making in nurseries.

**Abstract:**

Necropsies can reveal herd problems or comorbidities that can lead to management corrections, improvements in animal performance, and better decision making. Furthermore, the pattern and causes of mortality might differ when different systems are evaluated. The present study was conducted to establish the main causes of death in nursery pigs from different systems in Brazil, as well as the clinical, microbiological, and pathological aspects of these mortalities. Eighteen nurseries were analyzed (a total of 120,243 housed piglets), and 557 necropsies were performed. *Streptococcus suis* infection was the most prevalent cause of death (21.2%), followed by bacterial polyserositis (16.7%), chronic atrophic enteritis (13.5%), salmonellosis (8.8%), pneumonia (8.6%), and colibacillosis (6.1%). The increase in mortality rate in individual nurseries and, consequently, in the diagnoses was commonly associated with disease outbreaks. Infectious diseases constituted the largest portion of the diagnoses, making a great opportunity for improving production rates in herds. Moreover, the extensive range of observed diagnoses highlights the importance of conducting preliminary diagnostic investigations based on necropsy to determine the causes of death. This approach allows for the direction of complementary tests, which can diagnose agents with greater specificity. As a result, this allows for the implementation of more effective prevention and control strategies.

## 1. Introduction

Mortality in nursery pigs is an important cause of economic losses at this production phase and a valuable production efficiency indicator. The acceptable postweaning mortality rate in Brazil varies between 1 and 2%. However, disease outbreaks are not uncommon, and these rates can escalate up to five times [1]. In this regard, recent streptococcosis outbreaks have been observed in nursery pigs of different countries, including Brazil, with rates of mortality exceeding 18% [2]. Additionally, the economic impact caused by illnesses may be exacerbated by the occurrence of coinfections or immunosuppressive diseases, as demonstrated in porcine circovirus type-2 subclinical infections [3].

Nursery pigs are highly susceptible to bacterial and viral agents due to the numerous challenges they encounter during this phase. Important factors that contributed to the outset of diseases in nurseries include the decrease in passive immunity, a high number of origins (farrowing farms), exposure to new infectious agents, and adaptation to the environment and diet [4,5]. Therefore, it is expected that infectious diseases can represent a challenge in nurseries and increase mortality. Nevertheless, studies providing data on postweaning mortality, mainly focused on postmortem exams, are not well documented. Furthermore, the pattern and causes of mortality might differ when different systems are evaluated, underscoring the importance of conducting periodic investigations in herds [6].

Postmortem examination, initially through necropsy, is a common diagnostic method in veterinary medicine useful to investigate causes of death, although it is sometimes neglected. Necropsy is especially essential in situations of disease outbreaks and an increase in morbidity or mortality of unknown causes. Additionally, necropsies can reveal herd problems or comorbidities that may not clinically affect pigs but can result in management corrections, improvements in animal performance, and decision making [7]. Furthermore, a more accurate diagnosis is obtained by the association of auxiliary tools such as bacteriological, histopathological, and molecular examinations.

Brazil ranks as the fourth-largest producer and exporter of pork globally, with over 2 million breeding sows housed. The southern region of the country, comprising the states of Santa Catarina, Paraná, and Rio Grande do Sul, accounts for 71.94% of the total slaughtered pigs and 93.21% of national pork exports, with Santa Catarina leading in these percentages. Additionally, the state of Santa Catarina boasts the largest swine herd in Brazil, representing about 28.23% of the national herd [8].

Therefore, the present study aimed to establish the main causes of death in piglets from different nursery farms in Brazil as well as describe the clinical, microbiological, and pathological aspects associated with each disease diagnosed.

## 2. Materials and Methods

### 2.1. Study Design

The present study was conducted during the year 2022 in 18 nursery farms, named A to R, located in the state of Santa Catarina, Brazil. Each analyzed nursery unit housed piglets from the first to the last week of nursery (pigs from 21–27 to 63–70 days of age). Visits were conducted for five consecutive days in all nurseries. All pigs that died spontaneously or were euthanized by farm staff were analyzed, as they contributed to nursery mortality (euthanasia of piglets was a procedure carried out by some herds for only those debilitated animals and piglets in extremis that had no chance of clinical improvement or recovery). Clinical history and treatments administered were also recorded.

### 2.2. Postmortem Examination

A systematic necropsy was performed on all pigs that died. During the necropsy, the lungs were assessed for the extent of lesions using the pulmonary pneumonia index (PPI) [9]. Additionally, the body condition score was evaluated on a scale of 1–5, with 1: emaciated; 2: thin; 3: ideal; 4: fat; and 5: very fat [10]. For histological analysis, tissue samples (heart, lung, liver, spleen, kidneys, small intestine, large intestine, brain, spinal cord, urinary bladder, gall bladder, skeletal muscle, and lymph nodes) were collected and fixed in 10% formaldehyde solution. The organs were routinely processed and stained with hematoxylin and eosin (HE) and submitted to histopathological examination.

### 2.3. Bacteriological Analysis

Samples from lesions of different organs/tissues and/or exudates were collected and subjected to bacteriological analysis. Bacterial cultivation and characterization were performed as described previously [11]. Briefly, for samples of the respiratory system or systemic lesions (e.g., polyserositis, septicemia), enriched culture media (e.g., blood agar, chocolate agar) supplemented with factors X (hemin) and V (nicotinamide adenine dinucleotide-NAD) were used under an atmosphere of aerobic and microaerophilic conditions (aerobic + 5% CO_2_). For clinical samples from the digestive system, enriched culture media (e.g., blood agar) and selective differential media (MacConkey agar) were used under aerobic conditions, along with selective differential media (SPS agar) under microaerophilic conditions (aerobic + 5% CO_2_). Primary cultures were incubated and evaluated after 24, 48, and 72 h. In cultures with growth, colonies were subjected to biochemical analysis, serotyping, and/or genotyping to confirm the isolates.

In cases of suspected salmonellosis, the following steps were performed: pre-enrichment and selective enrichment, followed by plating on selective differential media. Biochemical characterization and serotyping of isolates were conducted according to the White–Kauffmann–Le Minor scheme [12].

### 2.4. Polymerase Chain Reaction (PCR) and Immunohistochemistry (IHC)

Clinical samples intended for the detection of specific agents through PCR were subjected to DNA or RNA extraction using a commercial kit (MagMAX™ CORE Nucleic Acid Purifications, Thermo Fisher Scientific, Waltham, MA, USA), following the manufacturer’s recommendations. The extracted RNA was submitted to the synthesis of cDNA with the enzyme GoScript^TM^ (Promega) and submitted to nested reverse transcriptase (RT)-PCR. PCR assay was performed for the detection of Porcine Circovirus type 3 (PCV3) [13] and Teschovirus [14].

Isolates of *Escherichia coli* were genotyped to determine pathotypes through the detection of fimbriae genes (F4, F5, F6, F18, F41) and toxins (STa, STb, Stx2e, LT, and EAST1) [15]. Additionally, *Streptococcus suis* [16] and *Glaesserella parasuis* [17] isolates were also genotyped to detect different serotypes. In summary, the isolates underwent DNA thermal extraction, quantification, assessment of extracted DNA quality, and dilution and were then subjected to the detection of target genes through multiplex PCR assay. Electrophoresis was performed using a 1.5% agarose gel with Brilliant Green™ (Miami Lakes, FL, USA) dye as the DNA intercalating agent.

Immunohistochemistry (IHC) was applied, when necessary, to paraffin-embedded tissues for cases with histological lesions compatible with porcine circovirus type 2 (PCV2) infection [18], septicemic salmonellosis [18], influenza A virus [19], and *Streptococcus suis* infection [20].

### 2.5. Bromatological Analysis of Feed and Soybean Meal

Bromatological analysis of the feed and soybean meal was conducted in cases where there was suspicion of an outbreak of dilated cardiomyopathy. The analysis included the determination of dry matter, mineral matter, ether extract, vitamins, protein, crude fiber, and solubility indices [21].

### 2.6. Statistical Analysis

The statistical analysis was performed using Statistical Analysis System software (SAS^®^, version 9.4—SAS Institute Inc., Cary, NC, USA). Age at death was classified into three groups: 21–35 days, 36–50 days, and 51–70 days. Frequency distributions of each diagnosis according to the classes of age were obtained through the FREQ procedure. Logistic regression models, using the GLIMMIX procedure, were used to compare the occurrence of each diagnosis among the classes of age at a 5% significance level. Descriptive results are shown as frequencies and percentages, depending on the variable.

## 3. Results

The number of housed piglets (size of nursery), average age housing, number of origins, type of production, and mortality rates for each nursery are presented in Table 1.

In the 18 nursery units analyzed, a total of 557 necropsies were performed. Among these cases, 93.2% (519/557) resulted in a conclusive diagnosis, while 6.8% (38/557) were inconclusive. Table 2 presents the frequency of the main diagnoses, along with the corresponding age data (mean, median, and age range for each condition), sex, and the farms where the diseases occurred. Infectious diseases constituted the most conclusive diagnoses, with a frequency of 72.9% (378/519), followed by undefined causes at 14.4% (75/519) (represented by the diagnostic group “chronic atrophic enteritis”), and non-infectious diseases at 12.7% (66/519).

Table 3 presents the distribution of cases for each established diagnosis within age ranges during the nursery phase. The data show a higher prevalence of mortality among nursery piglets aged 36–50 days in the evaluated nurseries, with 277 diagnoses in this age range. Bacterial polyserositis emerged as the most frequently diagnosed disease during this period (*p* = 0.003). Infections by *S. suis* were more prevalent between 51 and 70 days (*p* = 0.001), along with dilated cardiomyopathy (*p* = 0.006) and salmonellosis (*p* value: 0.014). Chronic atrophic enteritis exhibited greater occurrence between 21 and 50 days of age (*p* = 0.004), while death due to colibacillosis decreased throughout the nursery phase (*p* = 0.002). For other diagnoses, there were no statistical differences among the class of age (*p* ≥ 0.236).

Based on clinical history, gross and microscopic lesions, bacterial isolation, and auxiliary exams (PCR and IHC), the 14 main diagnostic groups mentioned above were formed as follows.

### 3.1. Group 1—Infections by Streptococcus suis (S. suis)

**Figure 1:**
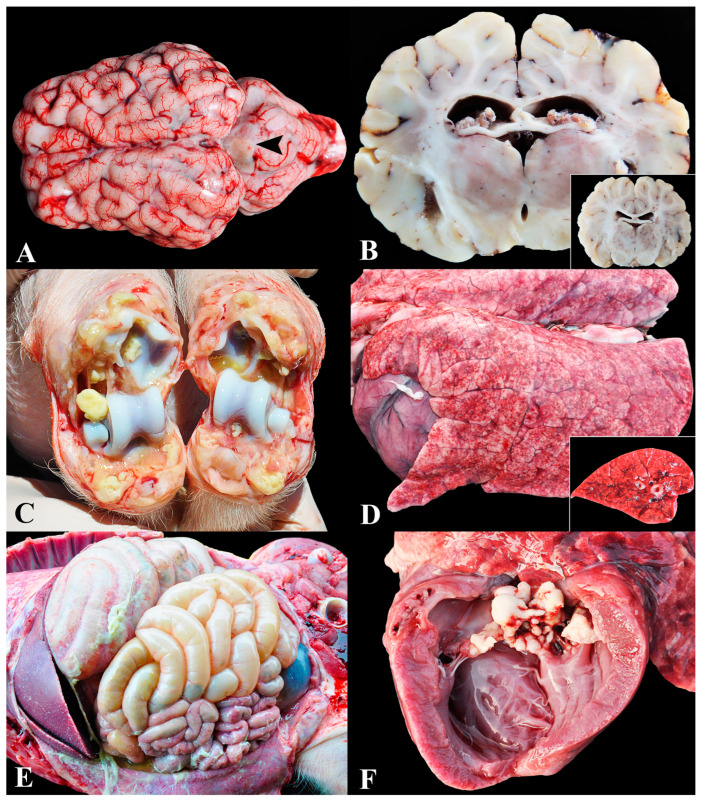
Infection by *Streptococcus suis* in nursery pigs: (**A**) Marked suppurative meningitis. Diffuse deposition of yellow material (pus) over the meninges (arrowhead) associated with marked hyperemia. (**B**) Fibrinosuppurative ventriculitis. Deposition of fibrin inside the lateral ventricles. Inset: Cross section of a normal brain for comparison. (**C**) Suppurative polyarthritis. Joints showing accumulation of purulent material in the articular capsules and joint cavities. (**D**) Interstitial pneumonia. The lungs are non-collapsed and elastic, exhibiting widespread petechial hemorrhages. Inset: Cross section of the lungs showing diffusely shiny and red parenchyma, along with enhancement of the interlobular septa by edema. (**E**) Fibrinosuppurative peritonitis. Accumulation of fibrin is noted on the serosa of the abdominal cavity organs. (**F**) Endocarditis. Irregular, white-yellow, and vegetative nodules adhered to the left atrioventricular valve.

The group named ‘*S. suis* infection’ was composed of pigs that exhibited suppurative/pyogenic lesions in one or more sites, accompanied by isolation of the agent in bacterial culture. Infections by *S. suis* were the predominant cause of death in this study, accounting for 21.2% (118/557) of cases. Gross and microscopic lesions consisted of meningitis (Figure 1A), encephalitis, and/or suppurative ventriculitis (Figure 1B) in 87.3% of piglets (103/118); suppurative polyarthritis in 59.3% (70/118), mainly affecting tarsal (Figure 1C), femoro–tibio–patellar, carpal, and humerus–radio–ulnar joints; lesions indicative of septicemia (such as interstitial pneumonia and splenomegaly) were present in 38.2% (45/118) (Figure 1D); fibrinous pericarditis in 9.3% (11/118); suppurative pyelitis in 8.5% (10/118); fibrinosuppurative polyserositis in 5.1% (6/118) (Figure 1E); endocarditis in 5.1% (6/118) (Figure 1F); and suppurative cystitis in 1.7% (2/118).

Significantly, most animals exhibited two or more of these lesions concurrently (88/118—74.6%). The main associations included meningitis and polyarthritis (53/118—44.9%); meningitis, interstitial pneumonia, and splenomegaly (44/118—37.3%); and meningitis, polyarthritis, interstitial pneumonia, and splenomegaly (25/118—21.2%). Clinical signs observed in streptococcosis cases included lameness, increased joint volume, recumbency, opisthotonos, paddling, nystagmus, seizures, fever, and sudden death.

Isolation of *S. suis* was successful in 79.6% of cases (94/118), with most isolates originating from brain samples (77/94). In 24 cases (24/118) where bacterial growth was not obtained, immunohistochemistry for *S. suis* was performed, resulting in positive findings for the agent in each sample (samples of brain, heart, and spleen). Additionally, in one nursery (nursery D), coinfection with Porcine circovirus type 2 (PCV2) was evident in six animals by IHC (6/22) with mortality related to streptococcosis. It was confirmed that a failure had occurred in the PCV2 vaccination protocol for this particular batch (no vaccination).

Samples that comprised all nurseries with streptococcosis diagnosis were serotyped, enabling the identification of the following serotypes: 2/½ (nursery F), 7 (nurseries J, K, L, M, and R), 9 (nurseries B, C, D, O, Q, and R), and 16 (nursery A). Samples from nurseries E and G were classified as non-typeable. In nurseries B, C, and D, the mortality rate was extremely high due to streptococcosis outbreak associated with serotype 9. Nursery K also presented outbreak mortality associated with serotype 7.

### 3.2. Group 2—Bacterial Polyserositis

**Figure 2:**
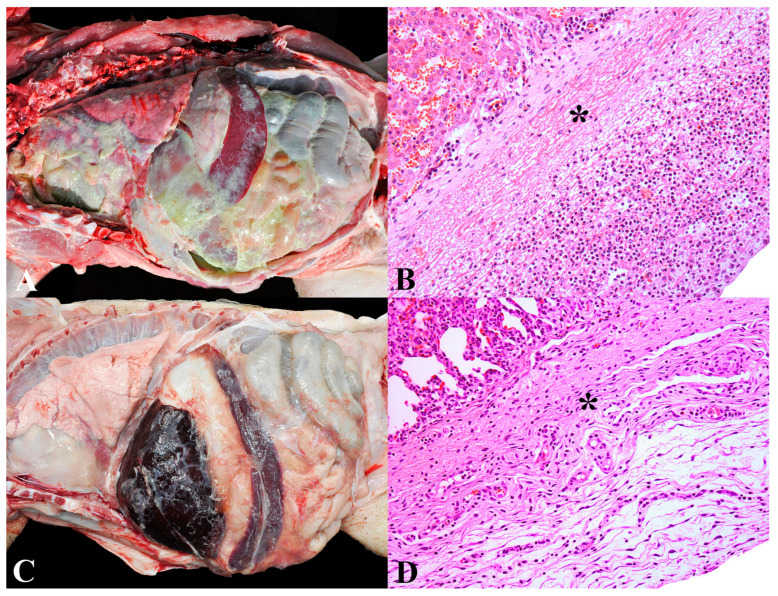
Bacterial polyserositis in nursery pigs: (**A**) Acute bacterial polyserositis. Marked deposition of fibrin on serosal surfaces of the abdominal and thoracic organs. (**B**) Acute bacterial fibrinosuppurative perihepatitis. Thickening of Glisson’s capsule due to fibrin deposition (asterisk) and infiltration of degenerated neutrophils. Hematoxylin and eosin, 200×. (**C**) Chronic bacterial polyserositis. Marked adherence of organs within the abdominal and thoracic cavities due to fibrous tissue proliferation. (**D**) Chronic bacterial pleuritis. Thickening of the pleura due to proliferation of fibrous connective tissue (asterisk) and blood vessels and mild infiltration of lymphocytes and macrophages. Hematoxylin and eosin, 200×.

Bacterial polyserositis represented the second most important cause of death in pigs in this study (93/557). The main clinical signs observed included wasting, dyspnea, increased joint volume, abdominal distension, weight loss, fever, seizures, and sudden death. This diagnosis was categorized into acute or chronic forms. Acute bacterial polyserositis was assigned to pigs with lesions in multiple serous membranes (pleura, pericardium, and peritoneum), joints, and/or meninges, accompanied by fibrinous exudate at these sites (Figure 2A,B). Cases with isolation of *S. suis* were not considered in this group as they were specifically grouped (Group 1).

The diagnosis of chronic bacterial polyserositis was assigned to animals exhibiting adhesions in serous membranes during macroscopic examination (pleura, pericardium, peritoneum), synovium, and/or meninges (Figure 2C). This was associated with the proliferation of fibrovascular connective tissue and a mixed inflammatory infiltrate, with a predominance of lymphocytes, macrophages, and plasma cells in microscopic lesions (Figure 2D). Bacterial isolation was not performed in chronic cases.

In total, 60 cases (60/93—64.5%) were categorized as acute (mean/median age: 42.7/43.5 days), while 33 (33/93—35.5%) as chronic (mean/median age: 49.9/49 days). In acute cases, successful isolation was made in 31.6% (19/60) of the samples, all of which were identified as *Glaesserella parasuis*. The isolates were predominantly recovered from the thoracic cavity (17/19), abdominal cavity (12/19), and pericardium (6/19). Among all cases of bacterial polyserositis, both acute and chronic forms, 79.5% (74/93) exhibited pulmonary histological lesions consistent with coinfection by the swine influenza virus (SIV), and 18.3% (17/93) had concurrent bacterial bronchopneumonia (mean PPI: 21.7%). In these cases of bronchopneumonia, isolation was achieved in 64.7% (11/17), with isolates of *Pasteurella multocida* (4/17), *Streptococcus suis* (4/17), and *Glaesserella parasuis* (3/17). Among cases of chronic bacterial polyserositis, 27.3% (9/33) of piglets exhibited lesions of congestive heart failure, five of which also exhibited hemoperitoneum due to hepatic rupture (5/9).

Additionally, serotyping of the samples identified serotypes 4 (nurseries B, C, and Q), 5 (nurseries A, E, F, G, H, and R), and 12 (nurseries K, L, M, and R). Samples of nurseries I, N, and O were classified as non-typeable. The nurseries L and Q showed an increase in mortality associated with an outbreak of polyserositis by *Glaesserella parasuis* serotypes 4 and 12, respectively.

### 3.3. Group 3—Chronic Atrophic Enteritis

**Figure 3:**
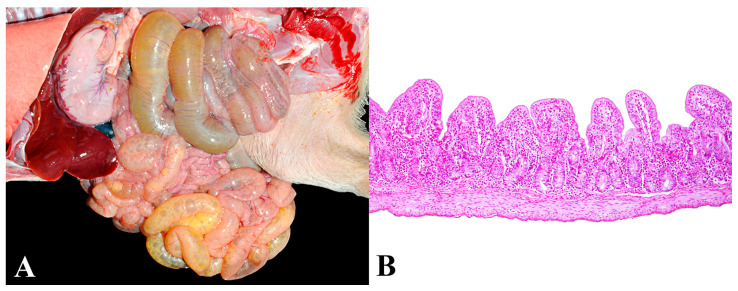
Chronic atrophic enteritis in nursery pigs: (**A**) Small intestine distended by a liquid content and thinning of the intestinal wall. (**B**) Histological segment of the jejunum showing marked villous atrophy. Hematoxylin and eosin, 100×.

The diagnosis was based on the gross observation of thinning of the intestinal wall, liquid and poorly digested intestinal content, emaciated to thin body condition score (<2.0), and obligatory histological lesions of severe villous atrophy (Figure 3A,B). In total, 75 diagnoses were obtained, with 82.7% (62/75) of the pigs exhibiting body condition scores as emaciated and 17.3% (13/75) as thin. Histologically, the presence of parasites of the Apicomplexa order within the cytoplasm of enterocytes was observed in four cases (4/75—5.3%), possibly *Cystoisospora suis*. No etiological agents were associated with villous atrophy in the remaining pigs within this group.

Other predominant histological lesions included macrovacuolar hepatic degeneration (54/75—72%) and renal tubular cell degeneration (17/75—22.7%). Additionally, deaths associated with chronic atrophic enteritis occurred in eight of the visited nurseries (8/18—44.4%), of which six (6/8—75%) had serious challenges of wasting and mortality due to rotavirus diarrhea in the originating farrowing units. However, a direct association of rotavirus in chronic atrophic enteritis in this study was not possible due to the chronic nature of the intestinal lesions. Therefore, this diagnostic group was considered an “undefined cause”.

### 3.4. Group 4—Salmonellosis

**Figure 4:**
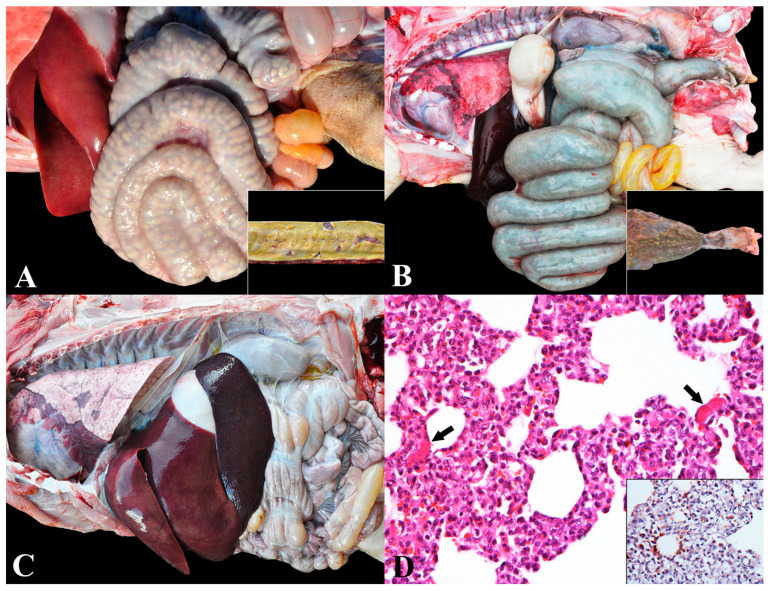
Salmonellosis in nursery pigs: (**A**) Enteric salmonellosis. Spiral colon with diffuse thickening of the wall and irregular surface (fibrinonecrotic colitis). Inset: jejunal fragment with exposed mucosa, showing fibrin deposition (fibrinonecrotic enteritis). (**B**) Chronic enteric salmonellosis. Megacolon associated with rectal stenosis secondary to chronic salmonellosis. Inset: the mucosa of the final part of the colon shows fibrin deposition (fibrinonecrotic colitis). A focal area of stenosis is seen in the rectum (rectal stenosis). (**C**) Septicemic salmonellosis. Marked hepatomegaly and splenomegaly. Non-collapsed lung (interstitial pneumonia) with cranioventral areas of atelectasis. (**D**) Interstitial pneumonia (septicemic salmonellosis). Lung with marked thickening of alveolar septa due to inflammatory infiltrate of lymphocytes, plasma cells, macrophages, and neutrophils associated with areas of microvascular thrombosis (arrow). Hematoxylin and eosin, 400×. Inset: multifocal immunolabeling in the cytoplasm of macrophages. Anti-*Salmonella* sp. immunohistochemistry, 400×.

Salmonellosis was the fourth most diagnosed disease (8.8%—49 cases), with lesions predominantly characterized by fibrinonecrotic typhlocolitis (37/49) and fibrinonecrotic enteritis (5/49—10.2%) (Figure 4A). Three chronic cases were identified, consisting of two cases of rectal stenosis and one of colon stenosis (Figure 4B). The disease was characterized as septicemic in 8.2% (4/49) of cases, as confirmed by immunohistochemistry (Figure 4C,D).

In nurseries H and M, enteric salmonellosis outbreaks occurred associated with *Salmonella* Typhimurium, which caused an increase in mortality rate (9.5 and 15%, respectively). Septicemic salmonellosis was observed in nursery M by the same serovar. Clinically, there were marked watery yellow diarrhea, dehydration, fever, and wasting in acute enteric salmonellosis. In septicemic form, the piglets also demonstrated cyanosis of the skin on the ears, snout, and legs, evidenced by purple-black discoloration. In contrast, in chronic salmonellosis, wasting of the piglets, abdominal distension, yellow-grayish diarrhea, and undigested feed in the colon were found.

### 3.5. Group 5—Pneumonia

**Figure 5:**
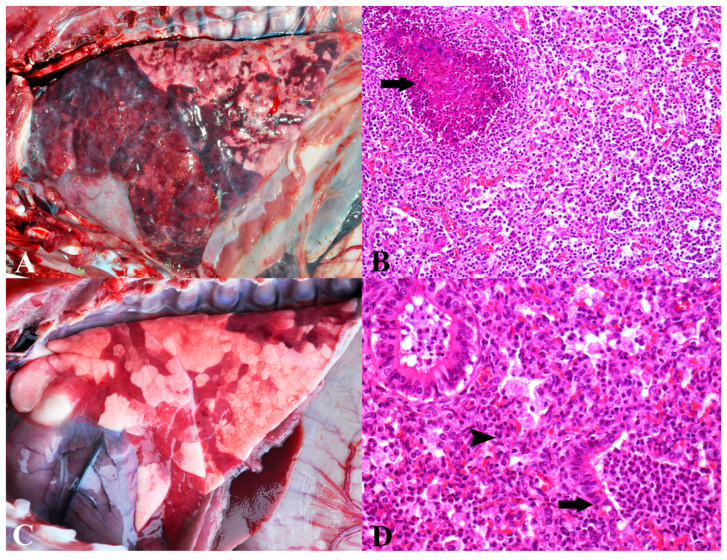
Pneumonia in nursery pigs: (**A**) Bacterial bronchopneumonia. Marked cranioventral lung consolidation. (**B**) Histologically, bronchioles are occluded by infiltrates of degenerated neutrophils and bacteria (arrow). In the parenchyma, there is also pronounced neutrophil infiltrate in alveoli. Hematoxylin and eosin, 200×. (**C**) Viral pneumonia (swine influenza). Non-collapsed lung with multifocal reddened and depressed areas of the parenchyma (atelectasis) (resembling a “chessboard” pattern). (**D**) In histology, bronchioles with infiltrate of neutrophils are associated with necrotic bronchiolitis (arrow). Additionally, there is interstitial lymphoplasmacytic and neutrophilic infiltration (interstitial pneumonia) (arrowhead). Hematoxylin and eosin, 400×.

Pigs included in this group exhibited extensive areas of pulmonary consolidation, affecting at least 30% of the parenchyma. Pneumonia was classified into viral and bacterial patterns. Bacterial pneumonia was characterized by cranioventral consolidation with dark-red discoloration, which sometimes extended to the caudal lobes (Figure 5A). Histologically, a marked degree of suppurative/fibrinopurulent/necrosuppurative bronchopneumonia or pleuropneumonia may be observed (Figure 5B). In viral pneumonia, the lungs were non-collapsed, smooth, and shiny, with pronounced multifocal areas of atelectasis, sometimes resembling a “chessboard” pattern (Figure 5C). In all cases, there were histopathological lesions of bronchointerstitial pneumonia with necrotic, proliferative, or obliterative bronchitis/bronchiolitis, characteristic of Influenza A virus infection (Figure 5D).

Pneumonia accounted for the fifth leading cause of observed death (8.6%—48 cases), with an average PPI of 49.7%. Among these, bacterial pneumonia made up 56.3% (27/48) of diagnoses, with histological lesions of concurrent viral pneumonia, indicative of SIV infection, present in 74.1% (20/27) of cases. Bacterial isolation was successful in 13 cases (13/27—48.1%), with isolates of *Pasteurella multocida* (7/13), *Streptococcus suis* (3/13), *Actinobacillus pleuropneumoniae* (2/13), and *Trueperella pyogenes* (1/13).

Viral pneumonia represented 43.7% (21/48) of diagnosed cases. No bacterial agents were cultured from lung fragments in these cases. All pneumonia cases had lung samples to IHC for SIV detection, resulting in five positive samples (5/48—10.42%). Another noteworthy aspect regarding pneumonia is that in 39.1% (199/509) of cases where animals died due to other causes, lesions compatible with viral pneumonia caused by SIV infection were observed. Additionally, none of the evaluated lung samples showed an indication of *Mycoplasma hyopneumoniae* infection.

Clinical signs included a previous history of respiratory distress as well as labored breathing, abdominal breathing, wasting, low weight gain, and sudden death. Some piglets were euthanized due to no clinical improvement by farm workers.

### 3.6. Group 6—Colibacillosis

**Figure 6:**
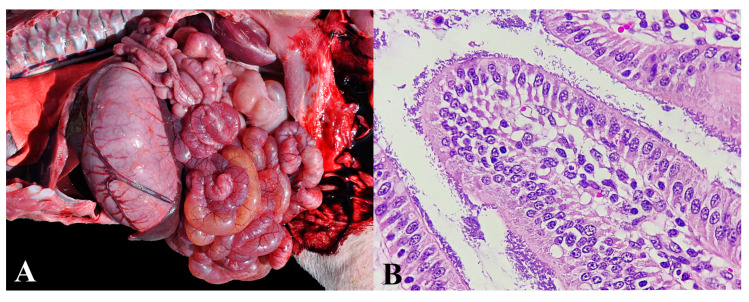
Postweaning colibacillosis in nursery pigs. (**A**) Hyperemic small intestine with intraluminal watery content. (**B**) Intestinal villus with a marked amount of coccobacillary bacteria closely adhered to the surface of enterocytes. Hematoxylin and eosin, 600×.

Infection by *E. coli* was diagnosed through the association of a clinical history of diarrhea, observation of distension of intestinal loops with watery content, intestinal hyperemia (Figure 6A), bacterial isolation, and microscopic lesions of cocobacillary bacteria adhered to enterocytes of intestinal villi (Figure 6B).

Bacterial isolation of beta-hemolytic *E. coli* was successful in all cases. Histologically, bacteria were visually detected in 52.9% (18/34) of the samples from the small intestine, often associated with intense hyperemia of mesenteric vessels. Samples with compatible clinical presentation but without histological observation of bacteria in loco were subjected to identification of the isolate’s pathotype to support the diagnosis. Among the genotyped *E. coli* pathotypes (16/34), all were identified as enterotoxigenic *E. coli* (ETEC). Fimbria F4 was observed in most cases (15/16), and fimbria F18 was observed in one case. The detected toxins were STa, STb, LT, Stx2e, and EAST1. Every strain presented at least two of these toxins, with the following toxin combinations observed: F4-STa-LT (4/16); F4-STa-STb-LT (3/16); F4-STa-STb-Stx2e (2/16); F4-STa-LT-Stx2e (2/16); F4-STa-STb-Stx2e (1/16); F4-STa-STb-LT-Stx2e (1/16); F4-STa-STb-LT-EAST1 (1/16); F4-STb-LT-EAST1 (1/16); and F18-STb-LT (1/16). An outbreak of colibacillosis occurred in nursery A, which increase the mortality rate.

### 3.7. Group 7—Dilated Cardiomyopathy

**Figure 7:**
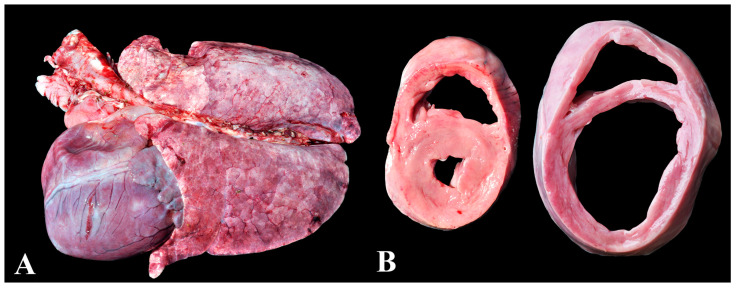
Dilated cardiomyopathy in nursery pigs: (**A**) Pronounced cardiomegaly, characterized by increased cardiac volume and loss of cardiac silhouette. (**B**) On the right, heart of a pig with dilated cardiomyopathy displaying marked dilation of the left ventricle associated with thinning of the ventricular wall when compared to the heart at the left (control).

Dilated cardiomyopathy accounted for the seventh cause of death (27/557—4.8%). This group comprised pigs that died suddenly and exhibited significant cardiomegaly (Figure 7A), with dilation of the cardiac chambers, either unilaterally or bilaterally, mainly in the ventricles (Figure 7B). Additionally, extracardiac lesions of congestive heart failure, such as ascites, edema, and pulmonary and hepatic congestion, were observed.

This diagnosis was obtained in four out of the 18 visited nurseries. One nursery (nursery P) experienced an outbreak of the disease, resulting in an 18% mortality rate, and most of the diagnoses occurred within this nursery (24/27). Analysis of the feed provided revealed low crude protein content and soybean meal solubility (crude protein: 40%; solubility: 62%). After replacing the feed with appropriate solubility, no further mortality related to this condition was observed. In the other three nurseries where the diagnosis was made, cases occurred sporadically, with one case in each nursery.

### 3.8. Group 8—Abdominal Organ Torsions

**Figure 8:**
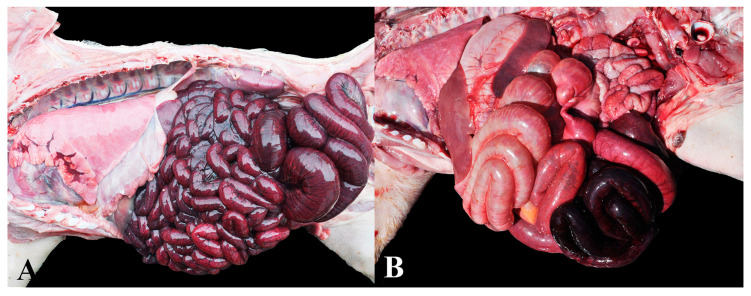
Organ torsions in the abdominal cavity in nursery pigs: (**A**) Counterclockwise 180° mesenteric torsion. Caudal displacement of the spiral colon associated with marked intestinal dilation by gas and diffuse dark red discoloration (congestion). (**B**) Segmental jejunal torsion (volvulus). Middle portion of the jejunum with pronounced congestion, dilation by gas, and devitalization of intestinal loops due to torsion.

This group consisted of cases in which gross observations revealed abdominal distension and torsion of the mesenteric root, segmental jejunal torsion, or splenic torsion. A total of 13 cases were diagnosed, including seven mesenteric torsions (Figure 8A) (median age of 58 days), five jejunal segmental torsions (Figure 8B) (median age of 40 days), and one splenic torsion (age of 43 days). All animals died suddenly and exhibited either an ideal or fat body condition score.

### 3.9. Group 9—Mulberry Heart Disease (MHD)

**Figure 9:**
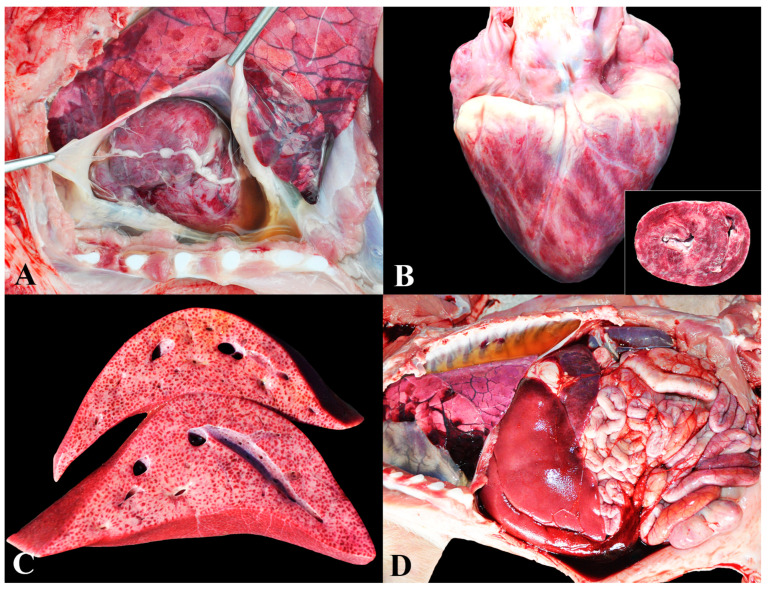
Mulberry heart disease in nursery pigs. (**A**) Mulberry heart disease. There is marked hydropericardium with fibrin filaments and epicardial hemorrhages. (**B**) Pronounced multifocal epicardial hemorrhages and slight presence of fibrillar material. Inset: On the heart’s cut surface, the hemorrhages extend into the myocardium. (**C**) Cut surface of the liver revealing a lobular pattern, with multifocal centrolobular reddened areas interspersed with pale parenchymal areas (centrolobular necrosis). (**D**) Abdominal cavity with a significant amount of free blood and blood clots (hemoperitoneum) due to hepatic rupture. Thoracic cavity with accumulation of citrine yellow fluid (hydrothorax), associated with non-collapsed and shiny lung, thickened interlobular septa (pulmonary edema), and multifocal red areas (hemorrhage).

MHD comprised diagnoses from three different nurseries involving piglets of various ages (age range: 29–50 days old). All pigs within this group exhibited cardiac lesions (Figure 9A,B) (13/13). Additionally, 53.8% (7/13) displayed liver necrosis and rupture associated with hemoperitoneum (Figure 9C,D).

Furthermore, vascular fibrinoid necrosis and leukoencephalomalacia were identified in a single case, occurring in the same piglet (1/13—7.7%). Other concurrent lesions included hydrothorax, hydropericardium, pulmonary edema, and hemorrhage. Notably, all pigs in this group died suddenly and displayed either ideal or fat body condition scores.

### 3.10. Group 10—Chronic Bacterial Pericarditis

**Figure 10:**
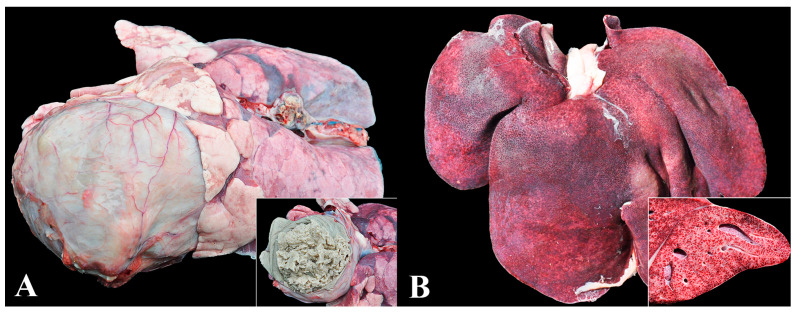
Chronic bacterial pericarditis in nursery pigs: (**A**) Bacterial pericarditis. Noticeable distension and thickening of the pericardial sac by a marked amount of fibrinous material (inset). (**B**) Liver presenting rounded edges, markedly congested, and displaying a lobular pattern, more evident on the cut surface (inset—“nutmeg” appearance), secondary to congestive heart failure due to chronic bacterial pericarditis.

This diagnostic group included cases in which there was a marked accumulation of exudate in the pericardial sac associated with chronic fibrinous pericarditis of likely bacterial origin (Figure 10A). Chronic pleuritis was also noted; however, no significant lesions were observed in other serous membranes. Additionally, there were extracardiac lesions of congestive heart failure, such as nutmeg liver (Figure 10B) and ascites. Bacterial isolation was unsuccessful in all cases (7/7).

All pigs had a previous history of antimicrobial treatment and no clinical improvement. Clinical signs included emaciation, abdominal distension, and respiratory distress.

### 3.11. Group 11—Teschovirus-Induced Polioencephalomyelitis

**Figure 11:**
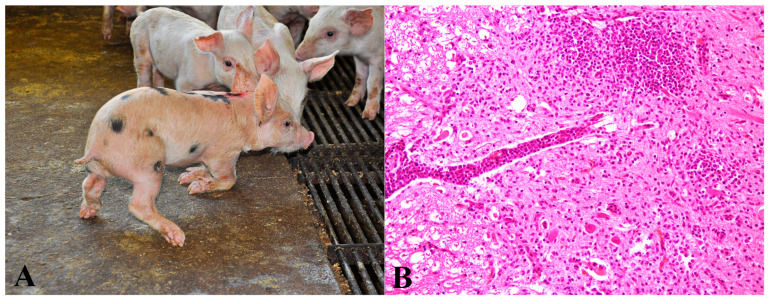
Teschovirus poliomyelitis in nursery pigs: (**A**) Clinical appearance of a pig with proprioception difficulty, walking on the hooves of the hind limbs and supporting the forelimbs on the carpal joint. (**B**) In the gray matter, pronounced multifocal inflammatory infiltrate of lymphocytes, plasma cells, and macrophages in the neuropil, sometimes surrounding blood vessels (perivascular cuffs), associated with neuronal necrosis and neuronophagia. Hematoxylin and eosin, 200×.

Pigs included in this group exhibited clinical signs of prolonged recumbency or motor incoordination (Figure 11A), along with histopathological lesions of non-suppurative poliomyelitis or polioencephalomyelitis associated with neuronal necrosis and neuronophagia (Figure 11B). Brain and spinal cord samples from all pigs were positive for Teschovirus in nested RT-PCR.

Teschovirus-induced polioencephalomyelitis affected six pigs, five of which were over 45 days old and originated from the same nursery (nursery Q). Among these cases, non-suppurative polioencephalomyelitis lesions were observed in four pigs (4/6—66.7%), while only non-suppurative poliomyelitis was present in two pigs (2/6—33.3%). Gross lesions were absent in all pigs.

### 3.12. Group 12—Porcine Circovirus Type 2 (PCV-2) Systemic Disease (PCV-2-SD)

Mortality associated with PCV2 infection was diagnosed in pigs with macroscopic and microscopic lesions consistent with the disease, along with positivity in immunohistochemistry (IHC) for the agent. All pigs in this group died suddenly and had an ideal body condition score.

**Figure 12:**
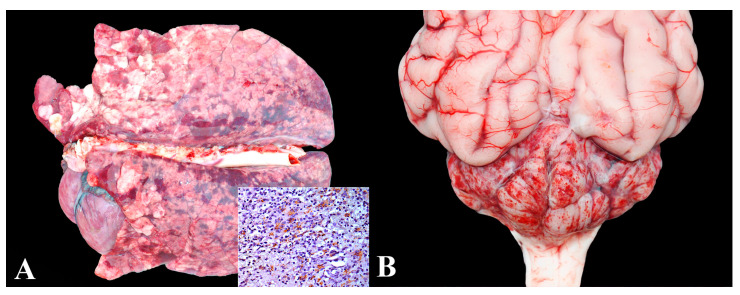
PCV-2-systemic disease in nursery pigs: (**A**) Non-collapsed and elastic lung with areas of consolidation, mainly in cranial lobes, as well as interlobular edema. Inset: marked multifocal immunolabeling in the cytoplasm of macrophages. Anti-PCV2 immunohistochemistry, 400×. (**B**) Cerebellar hemorrhage in PCV-2-SD. Notice the pronounced amount of petechiae in the cerebellar meninges.

Four pigs (4/5—80%) exhibited severe lesions of interstitial pneumonia (Figure 12A), granulomatous lymphadenitis, and multisystemic vasculitis, all originating from the same nursery (nursery R). Among these cases, two also displayed pronounced vasculitis and cerebellar hemorrhages (Figure 12B). The remaining pig (1/5—20%) (nursery Q) developed severe hepatopathy, characterized by marked hepatocyte necrosis and apoptosis leading to jaundice, along with vasculitis affecting multiple organs.

### 3.13. Group 13—Porcine Circovirus Type 3 (PCV-3) Systemic Disease (PCV-3-SD)

**Figure 13:**
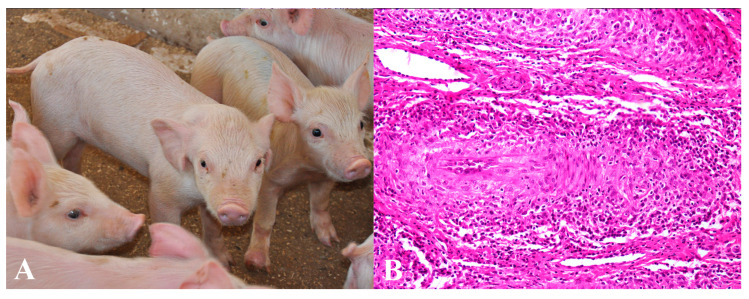
PCV-3-systemic disease in nursery pigs: (**A**) Characteristic clinical appearance of a “dumbo-like piglet” (PCV-3-SD), with ears rotated caudally. (**B**) Mononuclear perivasculitis in PCV-3-SD. Marked inflammatory infiltrate of lymphocytes, plasma cells, and macrophages is observed at the periphery of vessels in the mesenteric plexus (lymphoplasmacytic perivasculitis). Hematoxylin and eosin, 200×.

Pigs with mortality associated with PCV-3-SD were underdeveloped piglets (5/5—100%), with emaciated (2/5) to thin (3/5) body condition scores. These piglets displayed poor growth and weight gain and were often euthanized by the farm staff. All pigs had drooping and backward-turned ears, often referred to as “dumbo-like piglets” (Figure 13A), and exhibited pronounced histological lesions of systemic perivasculitis/mononuclear vasculitis (5/5) (Figure 13B), interstitial pneumonia (3/5), and mononuclear myocarditis (2/5). PCR results were positive for PCV3 in all piglets from lymph nodes, spleen, and lung samples.

### 3.14. Group 14—Bacterial Peritonitis Associated with Omphalitis

**Figure 14:**
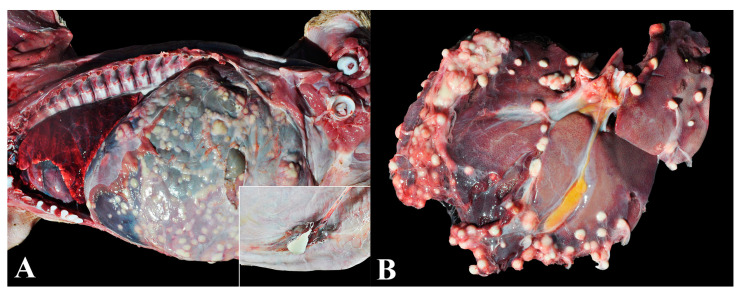
Bacterial peritonitis associated with omphalitis in nursery pigs: (**A**) Bacterial peritonitis associated with omphalitis. Multifocal abscesses in the serosal surfaces of abdominal cavity organs. Inset: Abscedative lesion in the umbilical cord. (**B**) Multiple abscesses of varying sizes adhered to the hepatic capsule.

The diagnosis of bacterial peritonitis was established in animals with pronounced lesions of abscedative peritonitis. The lesions were distributed throughout the peritoneum and extended to abdominal organs, primarily the liver, spleen, and intestines, invariably associated with abscedative omphalitis (Figure 14A,B). Bacterial isolation was successful in four cases (4/5—80%), with three cases showing isolations of *Trueperella pyogenes* and one case of *Pasteurella multocida* directly from abdominal cavity lesions (this case also presented mild multifocal embolic pneumonia). Piglets presented body condition score ≤ 2, emaciation, wasting, and abdominal distension.

### 3.15. Group “Others Conditions”

Other conditions encompassed less frequent diagnoses (21/557), including bacterial discospondylitis with spinal cord compression (4/21), chronic bacterial polyarthritis (4/21), coxofemoral luxation (3/21), intestinal intussusception (2/21), multicentric lymphoma (2/21), gastric ulcer (2/21), hemothorax secondary to pulmonary vessel rupture (1/21), rectal prolapse (1/21), vertebral column fracture (1/21), and urethral obstruction secondary to correction of an inguinoscrotal hernia (1/21).

## 4. Discussion

Industrial swine farming is characterized by confining numerous animals in proximity, which facilitates the transmission of infectious agents and the emergence of diseases [6]. Currently, infectious diseases remain the most prevalent issue in commercial pig farming in Brazil [22,23]. Furthermore, outbreaks of mortality are not uncommon in Brazilian nurseries, as our results have shown (outbreaks of streptococcosis, salmonellosis, colibacillosis, polyserositis by *Glaesserella parasuis*, and dilated cardiomyopathy). In this sense, it becomes clear that improvement in the mortality rate is a great economic opportunity in some production systems. The production cost can be high in the occurrence of clinical or subclinical diseases, as demonstrated in an experimental economic disease model of porcine circovirus type 2, with losses of up to GBP 84.1 per pig that died [3]. Moreover, research in wean-to-finish systems in the United States shows a reduction of USD 1.04 per head when mortality decreases from 6% to 5% [24].

However, postweaning mortality is multifactorial, and reducing the mortality rates is challenging. Many swine diseases are complex and triggered by interactions between non-infectious and infectious components, as seen in the porcine respiratory disease complex. Several factors related to management, environment, nutrition, disease occurrence, and proper diagnosis are important in this regard. Therefore, the accurate identification of the etiological agent, along with clinical-pathological aspects and, consequently, the disease involved, is a key point in decision making in herds [4,25].

*S. suis* infection represented the primary cause of death in nursery pigs in this study. This pathogen is responsible for various clinical–pathological manifestations in pigs, including arthritis, encephalitis/meningitis, polyserositis, pneumonia, endocarditis, and septicemia [26]. In our research, we observed all these alterations, often occurring in association with each other, and thus, we grouped them into a specific diagnostic category. Routinely, the disease has low occurrence in Brazil, with less than 5% mortality [27]. However, outbreaks of the disease have been occurring worldwide, particularly in nursery pigs, with high mortality rates attributed mainly to serotype 9 infections in Western Europe, Southeast Asia, and North America [28]. In Brazil, information regarding the characterization of *S. suis* serotypes related to clinical disease is still limited [27,29]. A study conducted by Matajira et al. [30], with isolates from clinical *S. suis* disease in Brazil between 2001 and 2016, demonstrated a higher prevalence of serotypes 2/½, 3, and 7, with no detection of serotype 9, which was also tested. However, more recent studies report outbreaks of the disease in Brazil associated with serotype 9, specifically in nursery pigs [2]. This was also noted in our study, where the mortality rates in three nurseries were higher due to the disease (15.7%, 17.0%, and 19.5% mortality) and associated with *Streptococcus suis* serotype 9. In one of the nurseries in the study (nursery D), *S. suis* infection was diagnosed concurrently with cases of circovirus infection due to vaccination protocol failure. This coinfection may have contributed to the worsening of the disease and increased mortality rates [22].

Bacterial polyserositis was the second most frequent diagnosis. In this work, *Glaesserella parasuis* was the only bacterium isolated, characterizing Glässer’s disease (GD). GD is a severe acute systemic disease that mainly occurs during the second week of housing in the nursery, between 35 and 50 days of age [31]. Exacerbation of GD cases is observed when there are concomitant infections with primary agents, such as swine influenza virus [32], as observed in 79.5% of the polyserositis cases in our study. Additionally, serotypes of high and moderate pathogenicity are also associated with worsening of GD, as identified in this study with serotypes 4, 5, and 12. However, recent research has demonstrated that serotypes with low pathogenicity can also cause serious infections [33]. Furthermore, the low sensitivity of bacterial culture for this agent is expected due to its fastidious growth characteristics, use of antibiotics, and autolysis grade of samples [34]. It is worth highlighting those other agents, such as *Pasteurella multocida*, *Mycoplasma hyorhinis*, and *Escherichia coli*, that can cause similar acute and chronic polyserositis in pigs [34,35].

Another significant group in this study was chronic atrophic enteritis, accounting for 13.5% of cases. Intestinal villous atrophy is a common pathological lesion that leads to poor nutrient absorption by enterocytes [36,37]. This condition is not necessarily fatal, especially when the lesions affect villous enterocytes rather than crypt-renewing cells [38]. In cases where intestinal lesions are mild and superficial, villous renewal can occur rapidly (12–24 h). However, in more severe cases involving the crypts, this process can take weeks or be persistent [36], leading to weight loss, cachexia, hypoalbuminemia, anemia, dehydration, diarrhea, and, in more severe cases, death [37]. Most pigs in the “chronic atrophic enteritis” diagnostic group came from farms where the farrowing origin had active rotavirus problems (6/8), which is the main associated pathogen in Brazil. However, it is difficult to directly associate chronic lesions with the virus. Additionally, a smaller number of pigs also had coccidiosis-associated enteritis (4/75—5.3%), which was possibly involved in some cases [37].

Cases of salmonellosis can occur in pigs of various ages, but they typically manifest within the first three or four months of age. Studies have demonstrated a high prevalence of active *Salmonella* sp. infection during the nursery phase [39]. Salmonellosis was the fourth leading cause of death in pigs from different systems evaluated in this study, highlighting the importance of this disease in the nursery phase, as observed in other recent studies in Brazil [40]. Additionally, a few cases of rectal and colonic stenosis were observed, which is a less common presentation of salmonellosis but is well documented in the literature [41]. Moreover, septicemic cases occurred in Nursery M, associated with the *Salmonella* Typhimurium serovar, which had a history of recurrent salmonellosis. Although *Salmonella* Choleraesuis is more commonly described in septicemic salmonellosis, *Salmonella* Typhimurium can also be involved [40].

Colibacillosis is a disease commonly observed in nursery piglets, especially within the first 2–3 weeks after housing, and it becomes uncommon after that period [42]. The pathotypes most associated with postweaning colibacillosis are typically enterotoxigenic (ETEC) beta-hemolytic colonies with fimbriae F4 or F18 and the production of one or more enterotoxins [43], as observed in this study.

Regarding pneumonia, swine influenza virus infection (SIV) has been demonstrated to have significant diagnostic importance. Since 2009, with the circulation of the pandemic H1N1 influenza A virus in Brazil, SIV has assumed a prominent role as a respiratory pathogen in pigs within the country [44,45]. Although it is now endemic, it acts as a primary agent of pneumonia in the Swine Respiratory Disease Complex (SRDC), enhancing the effect of secondary bacterial agents [22,46]. Due to its rapid passage through the respiratory tract, detection by PCR or IHC is often absent [19]. However, histopathological lesions in the lungs are typical and characteristic, serving as an important diagnostic tool [47,48]. The main opportunistic respiratory bacteria in Brazil are *Pasteurella multocida*, *Streptococcus suis*, *Actinobacillus pleuropneumoniae*, *Actinobacillus suis*, and *Trueperella pyogenes* [23]. *Pasteurella multocida* is the most isolated bacterium in swine bronchopneumonia in Brazil [23,49], as observed in our study.

Dilated cardiomyopathy was the primary cause of death related to non-infectious conditions. It is caused by a myocardium disorder that results in the dilation of the left or both chambers of the heart, leading to congestive heart failure and death [50]. The observation of a mortality outbreak in one of the evaluated nurseries influenced the number of diagnoses in this study. However, this emphasizes the potential significance of this disease as a cause of death in nursery pigs. Feed analysis revealed low levels of crude protein and soybean meal solubility. A similar nutritional etiology was identified in natural disease outbreaks in Brazil, which included experimental reproduction of the disease in nursery pigs [21]. Currently, the mechanism through which soybean meal with such altered characteristics triggers cardiac disorders is not understood. Dilated cardiomyopathy can also occur sporadically, affecting a small number of pigs, as observed in three other nurseries in the present study (nurseries H, Q, and R—one case each) and in another study [23]. In these isolated cases, it is challenging to attribute a specific cause to the disease. Recently, there has been a suggestion that the disease may have a genetic background in pigs due to a deficiency of δ-sarcoglycan, similar to what has been observed in humans [51].

Abdominal organ torsions can occur at all ages in pigs, but they are more commonly found during the growing and finishing phases [23], potentially contributing to up to one-third of mortality in these phases [52]. They have also been identified as significant causes of death in breeding sows, particularly liver lobe torsion [53]. Although the complete pathophysiology is not yet understood, risk factors associated with diet, management (restriction of feed followed by high consumption), and excessive fermentation of food content in the intestinal tract are described as predisposing factors [37].

Characteristic lesions of mulberry heart disease also constituted an important group in the study. Historically, deficiency of vitamin E and selenium has been associated with the development of the disease, which has already been reproduced experimentally. However, there are inconsistencies in the literature regarding this hypothesis, as similar levels of vitamin E and selenium have been found in the organs of animals with and without the disease [54,55]. Genetic predisposition is also suggested as a potential cause, although more research is required to fully understand this condition [55]. Pathologically, it can manifest in three main forms: mulberry heart disease, hepatic lipidosis, and nutritional myodegeneration, which can co-occur [56]. Other lesions, such as systemic fibrinoid vascular necrosis and leukoencephalomalacia, can also be found, as observed in one pig. This lesion is typically found in animals that survive for more than 24 h [56]. In association, hepatic rupture with hemoperitoneum was also observed (7/13). However, no studies have associated this disease with ruptures. One possible explanation for hepatic rupture is the pronounced congestion of the organ and centrolobular necrosis of hepatocytes, which result in an enlarged and friable liver leading to a rupture [57].

Chronic bacterial pericarditis accounted for 1.3% of the diagnoses. In pigs, this lesion is closely related to pneumonia and pleuritis, which can be disseminated through hematogenous spread or extend from pleural lesions [58]. The primary bacterial etiologies for pericarditis have been *S. suis*, *G. parasuis*, and *Actinobacillus* sp. [56]. Piva et al. [23] and Coelho et al. [58] also identified *Pasteurella multocida* as an important cause of bacterial pericarditis, either resulting in death or lesions found in abattoirs, respectively. Additionally, in the study carried out by Piva et al. [23], bacterial pericarditis accounted for 3.4% of mortality diagnoses in finishing pigs. All cases were also associated with congestive heart failure, and the isolation rate was low, possibly due to the chronicity of the lesions.

Viral polioencephalomyelitis in pigs can be caused by various agents, such as Teschovirus, Sapelovirus, and Astrovirus A, which induce similar lesions that are difficult to distinguish. Among these, Teschovirus is the most described in Brazil, initially identified in pig feces in 2015 [14] and later associated with clinical disease [59]. Currently, the virus is distributed worldwide and is considered endemic in pig populations; however, most cases are sporadic. Affected animals usually do not show clinical improvement, die from secondary causes, or are euthanized due to poor prognosis [60]. The diagnosis requires the association of histopathological lesions and agent detection in central nervous system samples using molecular techniques [61]. The lesions also frequently occur in the spinal cord, highlighting the importance of thorough spinal cord collection for an accurate diagnosis.

Systemic disease associated with porcine circovirus type 2 (PCV-2-SD) involves multiple organs and can result in highly variable gross lesions in different combinations. However, the histopathological lesions are characteristic and allow the confirmatory diagnosis if viral inclusions or high positive IHC staining is observed [62]. Currently, the agent is endemic in swine herds and is controlled through vaccination. The associated mortality is generally due to vaccine failure, possibly due to problems with vaccine administration, vaccinating animals outside the ideal age range, and potential inadequacy of vaccine immunity against viral variants [63].

PCV-3 is a recent etiological agent that has been reported in piglets. Although the pathogenesis of this virus is not yet fully clarified, previous studies have described cases of clinical diseases by the agent [13]. Recently, a proposal of PCV-3-SD was suggested based on the presence of clinical signs in affected animals, such as wasting and weight loss (criterion 1), marked systemic mononuclear perivasculitis infiltration (criterion 2), and PCV3 detection by quantitative PCR (qPCR) in the affected tissues (criterion 3) [64]. Additionally, in Brazil, a phenotype that has been molecularly diagnosed in PCV3-positive piglets is large and caudally rotated ears (known as “dumbo-like piglets”) [13]. According to this, our findings are consistent with PCV-3-SD; however, further studies are still needed to establish the impact of PCV-3 mortality in nursery piglets.

Secondary peritonitis resulting from lesions (e.g., ascending omphalitis, castration wounds, or bacterial enteritis/colitis) can occur in pigs, especially in the farrowing unit and nursery. These primary lesions can lead to focal abscesses in the umbilical region or ascend to the round ligament of the liver, urachus, and throughout the abdominal cavity, forming intracavitary abscessing lesions [65]. The main infectious agents associated with this condition are opportunistic bacteria, such as *Trueperella pyogenes*, *Staphylococcus* spp., *Streptococcus* spp., *E. coli*, and *Pasteurella* spp. [66].

## 5. Conclusions

The increase in mortality rate in individual nurseries and, consequently, in the diagnoses was commonly associated with disease outbreaks in this study. This was observed in cases of streptococcosis, polyserositis by *Glaesserella parasuis*, salmonellosis, colibacillosis, and dilated cardiomyopathy. Additionally, the extensive range of observed diagnoses highlights the importance of conducting preliminary diagnostic investigations based on necropsy and complementary tests (bacteriology, IHC, and PCR) to determine the causes of death. As a result, accurate diagnoses allow the implementation of more effective prevention and control strategies.

## Figures and Tables

**Table 1:** General data from eighteen nursery farms analyzed in Brazil.

Nursery	Month/Year	N° Housed Piglets	Average Age Housing (Days)	N° of Origins	Type of Production	Batch Mortality (%)
A	Mar/22	3500	22	1	Breeding herd + nursery ^3^	8.60%
B	Mar/22	2680	21	1	Breeding herd + nursery ^3^	15.70%
C	Mar/22	2500	21	1	Breeding herd + nursery ^3^	17%
D	Mar/22	3450	21	2	Nursery unit ^4^	19.50%
E	Apr/22	1613	27	4	Nursery unit ^4^	1.38%
F	Apr/22	6250	27	8	Nursery unit ^4^	3.16%
G	Apr/22	1288	27	3	Nursery unit ^4^	1.17%
H	Apr/22	2150	23	1	Breeding herd + nursery ^3^	9.50%
I	May/22	1450	21	1	Breeding herd + nursery ^3^	3.80%
J	May/22	2350	21	1	Breeding herd + nursery ^3^	4.20%
K	May/22	2900	21	3	Nursery unit ^4^	8.30%
L	May/22	4230	21	2	Nursery unit ^4^	6.50%
M	May/22	1750	21	5	Nursery unit ^4^	15%
N	May/22	3650	21	1	Breeding herd + nursery ^3^	3.90%
O	Jun/22	3250	24	1	Breeding herd + nursery ^3^	1.60%
P	Jun/22	3500	24	1	Nursery unit ^4^	18%
Q	Oct/22	26,644	23	1	Breeding herd + nursery ^3^	5.21%
R	Nov/22	47,088	24	65	Nursery unit ^4^	1.80%
-	-	120,243 ^1^	22.8 ^2^	102 ^1^		8.02% ^2^

^1^ Total; ^2^ average; ^3^ breeding herd + nursery: production system that handles the sow unit and nurse pigs; ^4^ nursery unit: receive and manage only weaned pigs.

**Table 2:** Postmortem diagnoses of nursery piglets from eighteen nurseries in Brazil.

Diagnosis	Total (%)	Sex (M/F)	Age Range (Days)	Average Age (Median)	Nurseries with Diagnoses
Infections by *S. suis* ^1^	118 (21.2)	72/46	22–64	49 (50)	A, B, C, D, E, F, G, J, K, L, M, O, Q, R
Bacterial polyserositis ^1^	93 (16.7)	46/47	27–70	45 (45)	A, B, C, E, F, G, H, I, K, L, M, N, O, Q, R
Chronic atrophic enteritis ^2^	75 (13.5)	33/42	25–60	42 (40)	A, C, H, M, N, O, Q, R
Salmonellosis ^1^	49 (8.8)	26/23	32–66	50 (50)	E, F, G, H, J, K, M, N, O, Q, R
Pneumonia ^1^	48 (8.6)	31/17	27–66	44 (45)	A, B, F, J, M, N, O, Q, R
Colibacillosis ^1^	34 (6.1)	13/21	28–51	37 (38)	A, C, H, I, J, N, Q
Dilated cardiomyopathy ^3^	27 (4.8)	17/10	35–70	55 (55)	H, P, Q, R
Abdominal organs torsion ^3^	13 (2.3)	6/7	27–60	48 (50)	B, E, I, J, N, Q, R
Mulberry heart disease ^3^	13 (2.3)	6/7	29–50	40 (42)	A, Q, R
Bacterial pericarditis ^1^	7 (1.3)	3/4	42–66	59 (60.5)	E, H, K, R
Teschovirus-induced polioencephalomyelitis ^1^	6 (1.1)	4/2	25–65	48 (49)	C, Q
PCV-2-systemic disease ^1^	5 (0.9)	2/3	44–66	55 (55)	Q, R
PCV-3-systemic disease ^1^	5 (0.9)	3/2	25–61	34 (29)	A, Q, R
Bacterial peritonitis (omphalitis) ^1^	5 (0.9)	3/2	37–60	48 (50)	E, F, P, Q, R
Other conditions ^1,3^	21 (3.7)	6/16	28–70	51 (50)	A, B, C, F, G, H, K, N, O, Q, R
Inconclusive	38 (6.8)	16/21	24–70	43 (43)	B, C, H, I, J, K, L, M, N, O, P, Q, R

^1^ Infectious causes; ^2^ undefined causes; ^3^ non-infectious causes.

**Table 3:** Postmortem diagnoses of nursery piglets from eighteen nurseries in Brazil, according to the classes of age.

Diagnosis	Percentage (Number) of Cases by Age Range (Days)			
	21–35 (Days) (*n* = 111)	36–50 (Days) (*n* = 277)	51–70 (Days) (*n* = 169)	*p* Value
Infections by *S. suis*	13.5% (15) b	18.4% (51) b	30.8% (52) a	0.001
Bacterial polyserositis	13.5% (15) ab	22.0% (61) a	10.1% (17) b	0.003
Chronic atrophic enteritis	19.9% (22) a	15.1% (42) a	6.5% (11) b	0.004
Salmonellosis	5.4% (6) b	6.9% (19) b	14.2% (24) a	0.014
Pneumonia	9.9% (11) a	9.8% (27) a	5.9% (10) a	0.333
Colibacillosis	13.5% (15) a	6.5% (18) b	0.6% (1) c	0.002
Dilated cardiomyopathy	0.9% (1) b	3.6% (10) b	9.5% (16) a	0.006
Abdominal organs torsion	0.9% (1) a	2.2% (6) a	3.5% (6) a	0.378
Mulberry heart disease	1.8% (2) a	3.2% (9) a	1.2% (2) a	0.369
Bacterial pericarditis	0.0% (0) a	0.7% (2) a	2.9% (5) a	0.236
Teschovirus-induced polioencephalomyelitis	0.9% (1) a	0.7% (2) a	1.8% (3) a	0.586
PCV-2-systemic disease	0.0% (0) a	0.7% (2) a	1.8% (3) a	0.611
PCV-3-systemic disease	3.6% (4) a	0.0% (0) a	0.6% (1) a	0.264
Bacterial peritonitis (omphalitis)	0.0% (0) a	1.1% (3) a	1.2% (2) a	0.994
Other conditions	4.5% (5) a	2.2% (6) a	5.9% (10) a	0.209
Inconclusive	11.7% (13) a	6.9% (19) ab	3.5% (6) b	0.035

Values with the same letter are not significantly different.

## Data Availability

The data sets and materials are available from the corresponding author upon reasonable request.
